# The influence of the COMT Val158Met polymorphism on prefrontal TDCS effects on aggression

**DOI:** 10.1038/s41598-024-53930-3

**Published:** 2024-02-10

**Authors:** Carmen Weidler, Lena Hofhansel, Christina Regenbogen, Dario Müller, Benjamin Clemens, Christian Montag, Andreas Reif, Ute Habel

**Affiliations:** 1 Department of Psychiatry, Psychotherapy and Psychosomatics, Faculty of Medicine, RWTH Aachen University, Aachen, Germany; 2 Institute of Neuroscience and Medicine: JARA-Institute Brain Structure Function Relationship (INM 10), Research Center Jülich, Jülich, Germany; 3 Department of Molecular Psychology, Institute of Psychology and Education, Ulm University, Ulm, Germany; 4 Department of Psychiatry, Psychosomatic Medicine and Psychotherapy, University Hospital Frankfurt, Frankfurt, Germany

**Keywords:** Cognitive control, Human behaviour

## Abstract

Increasing dorsolateral prefrontal cortex (DLPFC) activity by anodal transcranial direct current stimulation (tDCS) enhances cognitive control and might reduce aggression. The Val158Met polymorphism within the catechol-O-methyltransferase gene (rs4680) plays a pivotal role in prefrontal dopamine signaling, displaying associations with aggressive behavior, and potentially influencing the effects of tDCS. In a double-blind, sham-controlled study, we investigated the influence of rs4680 on tDCS effects on aggression. While undergoing functional magnetic resonance imaging, 89 healthy male participants performed the Taylor aggression paradigm before and immediately after tDCS. Actively stimulated participants (n = 45) received anodal tDCS (1.5 mA) for 20 min targeting the right DLPFC. Carriers of the val-allele (val+; n = 46; active tDCS n = 23) were compared to met-allele homozygotes (val−; n = 43; active tDCS n = 22). Analysis revealed decreased aggressive behavior in the val− group following active tDCS (*p* < 0.001). The val+ group showed increased aggression during the second session (*p* < 0.001) with an even higher increase following active as compared to sham tDCS (*p* < 0.001). No effects of stimulation or rs4680 on brain activation were found. Our study provides evidence for opposite tDCS effects on aggressive behavior in val-carriers and val-noncarriers. By shedding light on genetic factors predicting tDCS responsivity, the study will help to pave the way toward individualized—and thus more effective—tDCS treatment options.

## Introduction

Individuals with heightened or pathological aggression pose a substantial burden to society with severe consequences for health systems, economy and legal systems. While an increased likelihood of aggressive behavior is prevalent in many mental disorders, such as attention deficit/hyperactivity disorder (ADHD), substance use disorder, antisocial personality disorder or schizophrenia^1^, increased aggressive behavior is also observed in the absence of such conditions. Studies have shown that aggression is promoted by a multitude of psychological, environmental and biological factors. Examples from various description levels are e.g., dark personality traits^2^, altered brain function and genetic factors^3^.

Current studies investigating alterations in brain function have linked cortico-limbic dysfunctions to heightened aggression^3–5^. These dysfunctions are characterized by attenuated prefrontal control and altered activity of limbic regions. Among prefrontal regions, the right dorsolateral prefrontal cortex (DLPFC) has been shown to play a key role in the regulation of aggressive behavior^6,7^.

Besides these neural networks, there is robust data demonstrating that genetic factors play a role in heightened aggression. Heritability of aggression has been estimated to be around 50–60%^8^ but may be higher in individuals who exhibit high levels of aggressive behavior or high calleous-unemotional traits^9^. In line with the overall literature in psychiatric genetics, initial studies followed a candidate gene approach and the most frequently investigated genes are involved in the modulation of neurotransmitter levels, specifically serotonin and dopamine (DA). Accordingly, variations in the gene encoding for the enzyme catechol-*O*-methyltransferase (*COMT*), have been linked to aggressive behavior^10^. *COMT* plays an important role in the catabolism of catecholamines and crucially influences DA levels in the prefrontal cortex^11^. A functional single nucleotide polymorphism (SNP) in the *COMT* gene (rs4680) causes an amino acid substitution from valine to methionine at codon 158, resulting in a *COMT* phenotype with lower enzymatic activity and hence leading to higher DA levels in the prefrontal cortex^11,12^. This phenotype (met-allele carriers) has been associated with elevated aggression in patients with psychiatric disorders^13,14^ and healthy young individuals^15^. Furthermore, aberrant brain function has been linked to rs4680. Specifically, heightened reactivity of prefrontal and limbic brain regions during the processing of emotional stimuli has been reported for met/met homozygotes, suggesting a predisposition to emotional dysregulation in these individuals^16^. Meta-analytical evidence supports this assumption but has further linked the met-allele to more efficient prefrontal activation during cognitive processing^17^, suggesting a trade-off between adaptive and mal-adaptive consequences.

Despite the research performed to date, current pharmacological and psychotherapeutic interventions to decrease aggressive behavior remain insufficient. The apparent heterogeneity of aggression complicates treatment and demonstrates the need for personalized interventions such as non-invasive brain stimulation (NIBS) techniques including transcranial direct current stimulation (tDCS). TDCS allows researchers to modulate resting membrane potentials in targeted brain regions^18^. Physiologically, anodal tDCS increases the resting membrane potential, while cathodal tDCS decreases it^18^. Further research, however, has questioned this dichotomy and has claimed that at least for the modulation of cognitive functions, the relationship between current direction and (physiological) effects is not straightforward^19^. Furthermore, recent evidence has shown that tDCS effects on cognition are inconsistent and might be influenced by a number of individual factors, such as psychopathology, tobacco use, personality traits and genetic profiles^20–22^. Among genetic modulators of brain stimulation effects, rs4680 has been repeatedly investigated (for review see^21^). Interestingly, anodal stimulation of the left DLPFC impaired set-shifting abilities in homozygous carriers of the met-allele but not in carriers of the val-allele^23^. Correspondingly, cathodal stimulation of the same region impaired response inhibition in val-allele homozygotes but not in met-allele carriers^24^. Research suggests that the relation between DA levels and cognitive performance is best described by an inverted U-shaped function. Indeed, both very high and very low DA levels exert detrimental effects on performance^25^. In combination with prefrontal tDCS, individuals with the low activity *COMT* genotype (i.e. met-allele homozygotes) are thought to be more prone to detrimental effects of anodal stimulation, leading to excessive DA activity. Consistently, val-allele homozygotes will more likely show impairments of cognitive performance following cathodal stimulation, leading to insufficient DA activity. Moreover, studies have indicated that the interaction between tDCS and *COMT* genotype might be task-specific^21^.

To investigate the modulation of aggression using NIBS, most previous studies have applied anodal tDCS targeting the prefrontal cortex. Even though findings were inconsistent, a number of studies found beneficial effects of tDCS on aggressive behavior. A recent literature review on NIBS effects on aggression found significant downregulation of aggression through prefrontal tDCS in six out of 10 studies^26^. To date, however, there are no studies investigating the effect of rs4680 on tDCS effects on aggression.

With the current study we aimed to examine the effects of anodal tDCS over the right DLPFC in interaction with rs4680 on aggressive behavior in a sample of healthy males. In a double-blind, randomized, sham-controlled study, performance in a modified version of the Taylor Aggression Paradigm (mTAP) was assessed during functional magnetic resonance imaging (fMRI) before and immediately after a single session of tDCS. During the baseline measurement, we expected no difference in aggressive behavior between val-allele carriers and met-allele homozygotes. Following anodal, but not sham tDCS, we predicted beneficial stimulation effects in val-allele carriers and no or reversed effects in homozygous carriers of the met-allele.

## Methods

### Participants

A total of 89 healthy male participants took part in the study. Participants were German speaking, aged between 18 and 50 years (mean 25.73 ± 4.79) and right-handed. Exclusion criteria were any of the common contraindications for MRI and current neurological or psychiatric disorders. To guarantee an equal distribution of rs4680, individuals were genotyped prior to study participation. Similar to previous studies^23^, homozygous carriers of the met-allele (val−; n = 43) were compared to individuals carrying at least one val-allele (val+; n = 46). Participants were randomly assigned to receive either active or sham tDCS, resulting in the following subgroups: Val− active tDCS n = 22, val− sham tDCS n = 21, val+ active tDCS n = 23, val+sham tDCS n = 23.

### Genotyping

DNA was extracted from buccal swabs via a MagNA Pure 96 robot from Roche Diagnostics (Mannheim, Germany). Genotyping was done via melting curve analysis on a Cobas Z 480 analyzer from Roche Diagnostics (Mannheim, Germany). Simple probe assay designs were provided by TIBMolBiol (Berlin, Germany).

### Procedure

After giving the informed consent, participants were screened for current psychiatric disorders using the screening questionnaire for Axis I disorders of the Structured Clinical Interview for DSM Disorders (SCID I;^27^). Various questionnaires assessing aggression and impulsivity traits and neuropsychological tests assessing executive functions were completed (see below for details). Subsequently, participants were introduced to their (fictitious) opponent, a confederate of the experimenter, and they jointly listened to the instructions of the aggression paradigm. To circumvent priming and socially desired behavior, participants were told that the study assessed the effects of tDCS on attentional processes measured by a competitive reaction time task.

Following all instructions, participants completed a resting-state fMRI, and two paradigms during fMRI measurements, a modified version of the Taylor Aggression Paradigm and a stop signal task (SST; data not included here). The baseline scan was followed by the tDCS application outside the scanner during which participants performed a working memory task. Immediately after the termination of stimulation, participants returned to the scanner to again perform the mTAP, SST and structural MRI. At the end of the experiment, subjects were debriefed and compensated for participation. The study protocol was approved by the IRB of the medical faculty of the RWTH Aachen and in accordance with the Declaration of Helsinki.

### Questionnaires and neuropsychological tests

To assess personality traits linked to aggression and impulsivity, participants completed the German versions of the Buss-Perry Aggression Questionnaire (AQ)^28^, the Reactive Proactive Aggression Questionnaire (RPQ)^29^, the Barrett Impulsiveness Scale (BIS)^30^ and the Sensitivity to Punishment and Sensitivity to Reward Questionnaire (SPSRQ)^31^. To assess executive functions, participants completed the Trail-Making-Test A and B^32^, the digit span forward and backward^33^ and the Wortschatz-Intelligenztest to estimate crystallized verbal intelligence (WST)^34^. Questionnaire and neuropsychological test data is presented in Table 1.

**Table 1:** Sample characteristics.

Variable	Mean (SD)		*t*	*p*
	Val+(n = 46)	Val–(n = 43)		
Aggression Questionnaire (AQ)				
Total score	62.44 (12.42)	59.42 (11.65)	1.179	0.242
Anger	14.59 (4.20)	14.40 (4.62)	0.205	0.838
Hostility	16.78 (4.69)	16.00 (4.49)	0.803	0.424
Verbal aggression	13.52 (3.61)	13.98 (3.14)	− 0.63	0.529
Physical aggression	17.33 (4.98)	15.05 (4.05)	2.359	**0.021**
Reactive-Proactive Aggression Questionnaire (RPQ)				
Total score	7.84 (3.97)	7.23 (4.52)	0.675	0.501
Reactive aggression	6.51 (3.16)	5.61 (3.06)	1.365	0.176
Proactive aggression	1.33 (1.21)	1.63 (1.98)	-0.839	0.404
Cognitive Tests				
TMT-A (sec.)	21.23 (5.17)	20.34 (6.19)	0.727	0.469
TMT-B (sec.)	40.60 (13.29)	40.10 (14.49)	0.170	0.866
Digit Span	15.24 (3.47)	15.79 (3.79)	− 0.717	0.476
WST IQ	104.15 (10.40)	105.53 (9.79)	− 0.625	0.534
Barrett’s Impulsivity Scale (BIS)				
Total score	59.71 (9.91)	59.02 (7.70)	0.349	0.728
Sensitivity to Punishment and Reward Questionnaire (SPSRQ)				
Total score	19.44 (6.22)	17.05 (5.87)	1.813	0.073
Sensitivity to reward	11.12 (3.63)	10.44 (4.06)	0.808	0.421
Sensitivity to punishment	8.32 (5.10)	6.61 (4.05)	1.707	0.092
Modified Taylor Aggression Paradigm				
Mean punishment selection (baseline measurement)	55.47 (26.49)	53.04 (24.82)	0.447	0.656

SD = standard deviation; TMT = Trail-making test; sec = seconds; WST = Wortschatztest.

Significant values are in bold.

### Modified Taylor aggression paradigm (mTAP)

**Figure 1:**
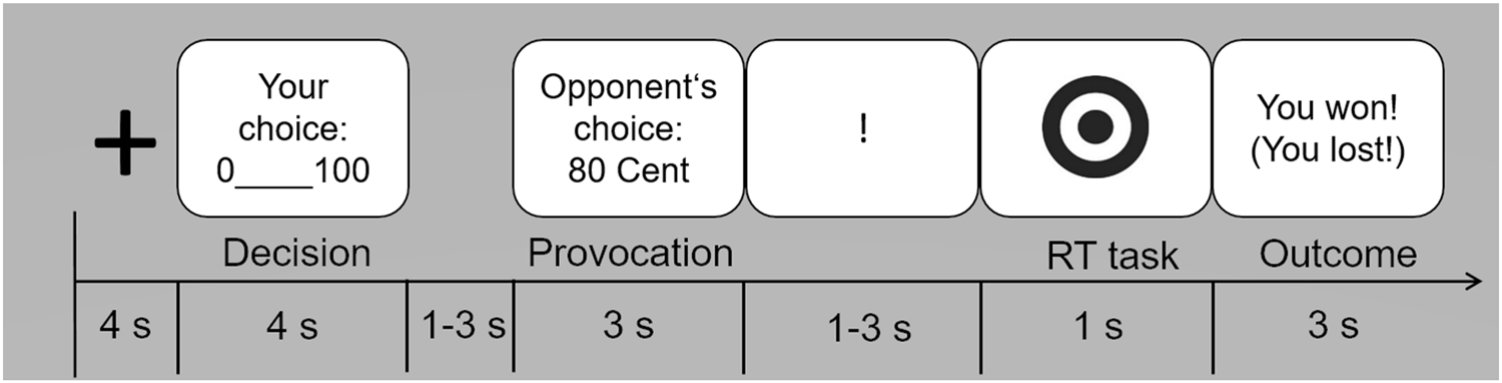
Depiction of a single trial of the modified Taylor Aggression Paradigm. Following a fixation cross, participants select a monetary punishment between 0 and 100 cents. Following a randomized jitter, participants are informed about the opponent’s punishment selection. A jittered presentation of the exclamation mark signals the upcoming reaction time task. Upon appearance of the visual target, individuals are asked to press a button as fast as possible. At the end of the trial, participants receive feedback on the outcome of the reaction time task.

At the beginning of each trial, individuals were able to choose a monetary punishment between 0€ and 1€ (decision phase). The decision phase was followed by the presentation of the opponent’s punishment selection (provocation phase). This was followed by the reaction time task during which participants were instructed to respond as fast as possible upon appearance of a visual target. The next screen presented the winner of the reaction time task (outcome phase). The sequence of a single trial is presented in Fig. 1. In total, participants completed three runs consisting of 30 trials each. Similar to previous studies^22,35–37^, provocation intensity increased from run one (range 0–40 cents) to run two (range 30–70 cents) and three (range 60–100 cents). A more detailed description of the paradigm has been previously provided^38^.

### TDCS

TDCS was delivered using a battery-driven stimulator (neuroConn, Ilmenau, Germany). Placement of the anode (5 cm × 7 cm) corresponded to the F4 position of the 10–20-EEG system. The cathode (10 cm × 10 cm), used as the reference electrode, was positioned over the contralateral supraorbital area with at least 7 cm distance to the anode. Following a 20 s ramp-up phase, participants in the active condition received a current of 1.5 mA for 20 min with a subsequent ramp-down phase of 20 s. In the sham condition, stimulation terminated after the ramp-up phase. The experimenter and the participant were blind to the type of stimulation.

### FMRI data acquisition

FMRI data was acquired using a Siemens 3 T Prisma scanner and a 20-channel head coil (Siemens AG; Erlangen, Germany). Functional images were collected using a spin-echo EPI sequence with the following acquisition parameters: TR = 2000 ms, TE = 28 ms, flip angle = 77°, voxel size = 3 × 3 × 3mm^3^, FOV = 210 × 210mm^2^, slice thickness = 3.3 mm, number of slices = 34 and matrix size = 64 × 64. For each run, 300 functional images were acquired in a descending order, resulting in a total of 900 functional images per participant. Structural scans were collected using a T1-weighted MPRAGE sequence with the following parameters: TR = 2300 ms, TE = 2.98 ms, flip angle = 9°, voxel size = 1 × 1x1mm^3^, FOV = 256 × 256mm^2^, slice thickness = 1 mm and number of slices = 176.

### Analysis of questionnaire and neuropsychological test data

Questionnaire and neuropsychological test data was compared between both *COMT* genotypes (Val+; Val–) using independent sample t-tests conducted in R (R Core Team, 2014). We included the total scores of the AQ, RPQ, SPSRQ and the BIS, as well as their respective subscales. Independent t-tests comparing cognitive performance included TMT-A (seconds), TMT-B (seconds), digit span (forward and backward), and verbal IQ as indicated by the WST.

### Behavioral data analysis

Data derived from the mTAP was analyzed using a linear mixed effects model on a trial-by-trial basis. Analysis was conducted in R (R Core Team, 2014) with the package *nlme*^39^. Random effect structures were modelled using the *lme4* package^40^. To find the best fitting structure, null models were compared using the *anova* function. *COMT* (val+, val−), tDCS (sham, active), time (pre stimulation, post stimulation) and provocation intensity from the previous trial were included as fixed effects. The interaction of COMT, tDCS and time was also taken into account.

### FMRI data analysis

Nine participants had to be excluded from the analyses due to insufficient data quality, resulting in an imaging dataset of 80 participants (val+active n = 21; val+sham n = 21; val− active n = 18; val− sham n = 20). Imaging data was analyzed with SPM12 software (Wellcome Department of Imaging Neuroscience, University College London, London, UK). Preprocessing involved realignment, segmentation, normalization and smoothing with a Gaussian kernel of 8 mm FWHM (Full Width Half Maximum; for details on the preprocessing see^38^. Following preprocessing, the three runs of the mTAP were concatenated for each individual. To account for the initial session length, the temporal non-sphericity calculations and the high-pass filter were corrected. On the first level, individual times series were fitted to a GLM including four regressors of interest. One regressor each modelled the decision and the provocation phase and two regressors the outcome of the reaction time task (win, lose). The anticipation of the reaction time game and the game itself were modelled by two regressors of no interest. Two parametric modulators were included to investigate brain regions showing response related modulations. The first parametric modulator was included for the decision phase, modeling the provocation intensity of the previous trial. The second parametric modulator was included for analysis of the provocation phase, modeling the provocation intensity presented to the participant. Six realignment parameters were included as regressors of no interest. Further, contrasts for differences between the first and second measurement were calculated for each condition of interest (decision, provocation, outcome). Separate full factorial models were calculated for the decision, provocation and outcome phase, each full factorial model including the between-subject factors tDCS (active, sham) and *COMT* genotype (Val+, Val−). Main effects of tDCS and *COMT* genotype as well as their interaction were inspected at FWE_cluster-level_* p* < 0.05. We further performed an exploratory region of interest (ROI) analysis to investigate possible differences in neural processing beneath the anode. The corresponding anatomical ROI (right middle frontal gyrus; MFG) was defined using the Wake Forest University (WFU) PickAtlas toolbox for SPM. Beta values for the parametric modulation of provocation during the decision phase and during the provocation were extracted for each individual within the right MFG. Two separate linear models were estimated using R including the fixed factors tDCS (active, sham) and *COMT* (val+, val–).

## Results

### Questionnaire and neuropsychological test data

Comparison of questionnaire and neuropsychological test data yielded only a significant higher physical aggression subscale of the AQ in the val+ as compared to the val− group (*t*(87) = 2.36, *p* < 0.05). This result did not withstand correction for multiple comparisons using Bonferroni correction. Detailed results on all comparisons can be found in Table 1.

### Behavioral analyses

For the linear mixed effects model, parameter estimates for fixed effects on punishment selection are presented in Table 2. The estimated effect size of the model was R^2^_Conditional_ = 0.62. Random effects included the intercepts and slopes per subject influenced by trial (1–90) and time (pre, post), and random slopes for subjects influenced by provocation (1–100). The linear mixed effects model revealed a main effect of time (*t*(15,749) = -9.76, *p* < 0.001), showing higher punishment selections in the second session. Results further indicated a main effect of provocation, resulting in higher punishment selections following increased provocation intensity (*t*(15,749) = 12.24, *p* < 0.001). A two-way interaction effect of time and *COMT* (*t*(15,749) = 10.37, *p* < 0.001), and a three-way-interaction between time, tDCS and *COMT* ((*t*(15,749) = 7.80, *p* < 0.001) yielded significant results.

**Table 2:** Fixed effects of linear mixed effects model.

Predictor	β	SE	Lower-95%	Upper-95%	*t*	*p*	*df*
Intercept	53.59	2.40	48.90	58.29	22.37	< 0.001	15,749
Time	− 1.78	0.18	− 2.14	− 1.42	− 9.76	< 0.001	15,749
tDCS	− 2.79	2.39	− 7.54	1.95	− 1.17	0.245	85
COMT	2.33	2.39	− 2.41	7.08	0.98	0.331	85
Provocation	12.56	1.03	10.55	14.58	12.24	< 0.001	15,749
Time x tDCS	0.08	0.18	− 0.28	0.43	0.42	0.674	15,749
Time x COMT	− 1.89	0.18	− 2.25	− 1.54	− 10.37	< 0.001	15,749
tDCS x COMT	0.07	2.39	− 4.68	4.81	0.03	0.977	85
Time x tDCS x COMT	1.42	0.18	1.07	1.78	7.80	< 0.001	15,749

β = regression coefficient; SE = standard error, Lower-95% = lower limit of 95% confidence interval, Upper-95% = upper limit of 95% confidence interval, df = degrees of freedom; tDCS = transcranial direct current stimulation; COMT = catechol O-methyltransferase.

**Figure 2:**
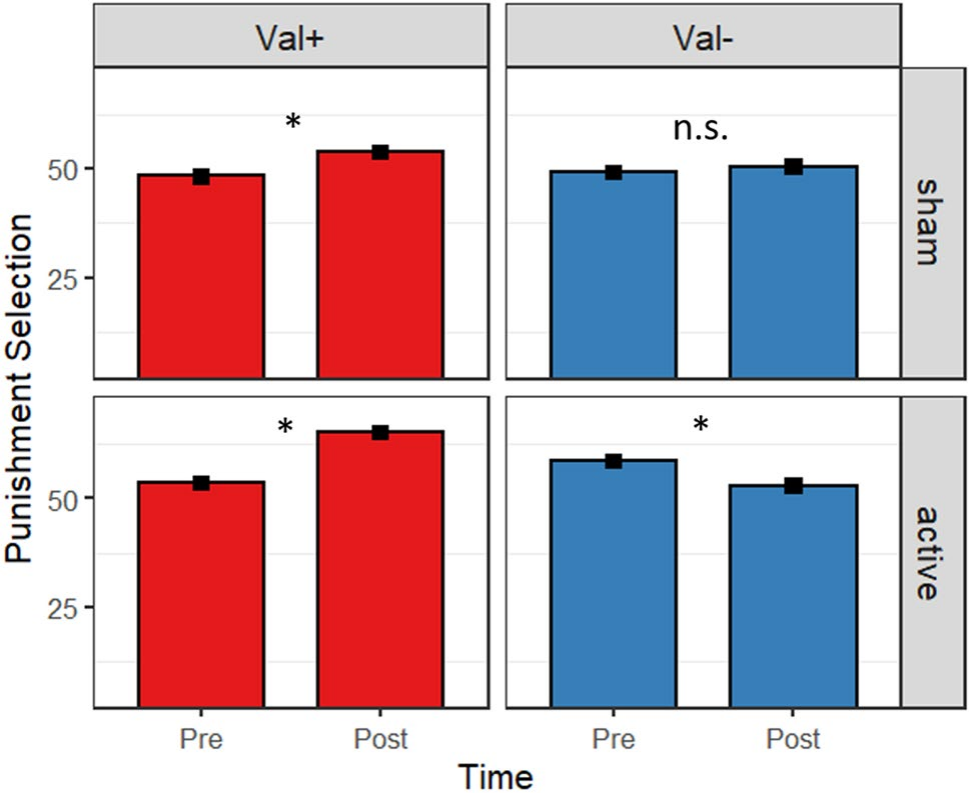
Behavioral results of the linear mixed effects model for the modified Taylor Aggression Paradigm. Punishment selections (Cents) are shown for carriers of at least one val allele (Val+; left panel) and homozygous met allele carriers (Val−; right panel) before (pre) and after (post) receiving either sham (top panel) or active (bottom panel) stimulation. The val+ group showed a significant increase in punishment selection from first to second session, independent of the stimulation condition (p < 0.001). Furthermore, the increase was significantly higher in the active as compared to the sham group (p < 0.001). The val- group receiving sham stimulation did not show any difference in punishment selections, while those receiving active stimulation showed a significant decrease in punishment selections in the second session (p < 0.001). Error bars represent standard error. Post hoc pairwise comparisons are corrected for multiple comparison using the Tukey method. Val+ n = 46, active tDCS n = 23; Val− n = 43, active tDCS n = 22; *p < 0.001.

Post hoc pairwise comparisons showed that the val + group, independent of stimulation condition, selected higher punishment levels in the second session (*p* < 0.001). Next, we compared the increase in punishment selections from pre to post of the val + group receiving sham to the val- group receiving active stimulation. The contrast revealed that the val + group showed a significantly higher increase in punishment selections following active as compared to sham stimulation (*p* < 0.001). Post hoc pairwise comparisons further showed that the val- group significantly decreased punishment selections in the second session following active stimulation (*p* < 0.001), while individuals receiving sham stimulation showed no significant difference in punishment selections between both sessions. Results of all comparisons are presented in Table 3. The three-way interaction is depicted in Fig. 2.

**Table 3:** Post-hoc pairwise comparisons of significant interactions.

Interaction effect	Contrast	*b*	*SE*	*t* ratio *p*	
Time x COMT	Val+_pre–post_	− 7.346	0.482	− 15.233	< 0.001
	Val−_pre–post_	0.224	0.548	0.409	0.977
	Pre_val+–val-_	0.883	4.786	0.184	0.998
	Post_val+–val-_	8.453	4.786	1.766	0.297
Time x tDCS x COMT	Pre, val+_sham–active_	− 2.453	6.413	− 0.383	0.999
	Pre, val–_sham–active_	− 8.415	7.107	− 1.184	0.934
	Pre, sham_val+–val-_	3.863	6.907	0.559	0.999
	Pre, active_val+–val-_	− 2.098	6.628	− 0.317	1.000
	Val+, sham_pre–post_	− 4.348	0.739	− 5.884	< 0.001
	Val + , active_pre–post_	− 10.345	0.620	− 16.687	< 0.001
	Val–, sham_pre–post_	− 2.467	0.851	− 2.898	0.0730
	Val–, active _pre–post_	2.916	0.690	4.225	< 0.001
	Val+, sham _pre–post_–Val+, active_pre–post_	6	0.964	6.219	< 0.001

*p*-values adjusted for multiple comparisons using the Tukey method; SE = standard error; tDCS = transcranial direct current stimulation; COMT = catechol O-Methyltransferase.

### Imaging analyses

Neither full factorial model revealed any suprathreshold activation (FWE_cluster-level_ *p* < 0.05) for main effects of tDCS and *COMT* or their interaction. Average effects of the decision and provocation phase for pre and post stimulation sessions are provided in the supplementary material (supplementary Figs. S1 and S2). ROI analysis (right MFG) did not yield any significant effect of tDCS (*p* = 0.258), *COMT* (*p* = 0.602) or their interaction (*p* = 0.152) on brain activity during the decision phase (contrast post–pre). Similarly, no significant effect of tDCS (*p* = 0.228), *COMT* (*p* = 0.644) or their interaction (*p* = 0.610) was found on brain activity during the provocation phase (contrast post–pre).

## Discussion

The current study aimed to investigate the effects of prefrontal brain stimulation and the *COMT* val158met polymorphism on aggressive behavior and the corresponding neural correlates in healthy males. As a measure of aggression, participants performed the mTAP during fMRI measurements before and immediately after receiving either active or sham tDCS. We could show that rs4680 is associated with aggressive behavior following repeated provocation and responsiveness to anodal tDCS of the right DLPFC. We could not observe any effect of tDCS, *COMT* genotype or their interaction on brain activity during the aggression paradigm.

Aggressive behavior, as measured by the mTAP, was strongly predicted by the preceding provocation intensity, replicating previous findings^22,35,37,41^. The association of rs4680 with aggressive behavior seems complex. While we did not observe any baseline differences between genotypes, punishment selections differed in the second session in that val-allele carriers showed a significant increase in aggressive behavior independent of stimulation condition, whereas no changes of behavior were observed in met-allele homozygotes following sham stimulation. Although we expected an increase in punishment levels from session one to two in the sham condition^22^, the absence of this increase in met-allele homozygotes was surprising, contradicting the frequently reported association of the low-activity *COMT* met-allele with increased aggression^13–15,42^. More consistent with our findings is the association of the val-allele with poorer response inhibition reported for patients with ADHD^43^. Furthermore, a meta-analysis of genetic association studies could not confirm a general effect of the *COMT* val158met polymorphism on aggression^44^. Thus, baseline differences in prefrontal DA tone, which might be different in healthy controls and psychiatric populations, might result in differential effects of *COMT* rs4680. Aligning well with our results and the evidence for increased DA levels in met allele carriers^45^, results of a PET study in healthy men suggest a protection against aggressive responses to provocation by greater DA availability^46^. Specifically in the regulation of aggressive responses to provocation, both, emotion regulation and cognitive abilities play important roles. Studies suggests that emotional and cognitive processes might be differentially impacted by the val158met polymorphism^16,17^, further complicating the interpretation of our results. Taken together, current findings suggest complex effects of rs4680 on aggression that are likely influenced by further variables such as other genotypes affecting the dopaminergic system, environmental factors, sex or age^47^. Also, candidate gene approaches such as the present one are susceptible to false positive findings, especially in smaller samples, and recent studies demonstrated frequent non-replication in genome-wide association studies (GWAS). Thus, future studies might benefit from either completely unbiased GWAS approaches using polygenic risk scores (PRS), or alternatively gene-based or gene-set-based PRS on genes encoding for the DA system^48,49^.

### Influence of the COMT val158met polymorphism on tDCS effects

Contrary to our hypothesis, we found that individuals with high DA availability due to low functioning *COMT* (val-) respond with decreased aggression to tDCS as opposed to val-allele carriers. The lack of increased aggression in met/met-allele carriers in the second session was paralleled by decreased aggressive behavior following anodal tDCS of the right DLPFC. Active stimulation in val-allele carriers had the opposite effect, resulting in a significant higher increase in aggressive behavior following active as compared to sham tDCS. A dampening effect of tDCS on aggressive behavior in general, is in line with a recent review reporting significant downregulation of aggression by tDCS in six out of 10 studies^26^. Moreover, numerous studies report differences in responsiveness to tDCS effects on cognition for specific *COMT* genotypes (reviewed by^21^). Disadvantageous effects of prefrontal anodal tDCS in met-allele carriers^23^ and worsening of cognitive performance in val-allele carriers following cathodal stimulation^24^, support the notion of an inverted U-shaped function describing optimal DA levels. Anodal tDCS in met-allele carriers is assumed to push DA levels beyond optimal levels, possibly explaining disadvantageous effects. In line with these studies, older adults carrying the high activity val-allele showed higher responsiveness to tDCS-induced cognitive enhancement as compared to met-allele homozygotes^50^. Importantly, a significant interaction of stimulation and *COMT* genotype was not evident in all cognitive tests assessed by studies described above, pointing towards a task-specific interaction^21^, possibly also explaining the opposite effects found in the current study.

Next to task-specificity, differences in the modulatory effects of the *COMT* genotype on tDCS effects could also arise from online and offline stimulation designs. The studies previously reported investigated the interaction of *COMT* and tDCS effects during the critical task, whereas rather few studies investigated the influence of the val158met genotype on tDCS after-effects. While modulated cortical excitability is thought to underlie online tDCS effects, the after-effects have shown to rely on changes in neural plasticity^18,51^. A recent study reported that tDCS after-effects on working memory were not modulated by the *COMT* genotype^52^. Notably, all mentioned studies investigated *COMT* genotype effects on tDCS modulation of cognitive performance and research focusing on this interaction in different contexts is scarce. One recent study, also focusing on tDCS after-effects, observed an interaction of anodal tDCS and *COMT* val158met polymorphism on the regulation of appetite. Consistent with our results, prefrontal tDCS led to reduced appetite in met-allele carriers and a rise in appetite in val-allele homozygotes^53^. The authors hypothesized that these individuals were subjected to large, tDCS-related fluctuations in DA, which might have caused abnormal reward processing. This is consistent with animal studies showing that tDCS can increase DA release^54^. The opposing effects of anodal tDCS in *COMT* genotypes reported by this study are strikingly similar to our own results. The regulation of appetite and aggression both rely on DA-modulated brain circuits and comprise similar neural networks involved in executive control, reward processing and self-directed thinking^41,55,56^. It is conceivable that similar mechanisms lead to these opposed tDCS effects in *COMT* genotypes, however, the exact mechanisms of these interactions remain poorly understood. While optimal DA levels might be necessary for beneficial tDCS effects on aggressive behavior (as seen in met-allele homozygotes), it remains unclear why anodal tDCS has the opposite effect on val-allele carriers, leading to an even higher increase in aggressive responses than sham stimulation. Nevertheless, it becomes evident that the effect of the *COMT* val158met polymorphism on tDCS effects is not straight forward, and can potentially lead to an inversion of the intended stimulation effect. This has noteworthy implications for the potential application of tDCS in clinical settings, emphasizing that a universal protocol is not applicable. This underscores the crucial need for personalized stimulation protocols, which incorporate individual factors such as genetic profiles.

As previously mentioned, we could not detect any influence of the *COMT* genotype or tDCS on brain activation. Although imaging studies have reported differences in brain function in emotion regulation and cognitive processes between genotypes, studies investigating the interaction of the *COMT* genotype and tDCS were purely behavioral. Hence, we cannot relate our null findings to existing studies. It is conceivable that this lack of differences in brain activation is related to the study design. As the aggression paradigm was performed offline, e.g. following the termination of stimulation, we are not able to examine online tDCS effects on brain activation. With our sample, we might also be underpowered to link brain function and behavior^57,58^.

### Limitations

Although the sample size might be small for the investigation of effects of genetic variants, behavioral results of the current study still reveal an interaction of the *COMT* val158met polymorphism and tDCS effects on aggressive behavior. However, we were not able to detect neural correlates of these effects, which calls for further investigation. Furthermore, with the methodology used in this study, we are not able to evaluate mechanisms underlying the influence of the *COMT* val158met polymorphism on tDCS effects. Future studies should implement simultaneous tDCS-fMRI measurements to directly investigate the contribution of *COMT* genetic variants to the variability in neural correlates of online tDCS effects. While the *COMT* val158met polymorphism has been repeatedly found to influence tDCS responsiveness, there are other genetic variants that affect DA signaling that were not assessed in the current study. Additionally, aggression is affected by further (biological) factors such as the serotonergic system^3^. Furthermore, we limited our sample to men. Consequently, we cannot extend our results to women. The literature indicates that gender can affect both, the responsiveness to tDCS and the effects of *COMT* genotypes on aggressive behavior^59–61^.

## Conclusion

In conclusion, our study demonstrates for the first time that differences in the *COMT* val158met polymorphism modulate the effects of prefrontal tDCS on aggressive behavior. The fact that tDCS can have the opposite of the intended effect underscores the significance of individual factors. This highlights the necessity for additional investigations to refine and personalize stimulation protocols, particularly in the context of clinical applications.

### Supplementary Information

Supplementary Information.

## Data Availability

The dataset analyzed during the current study is not publicly available because we do not have the ethics votum for sharing the data but are available from the corresponding author on reasonable request.
