# Supplementary material for: The influence of the COMT Val158Met polymorphism on prefrontal TDCS effects on aggression
Source: Sci Rep. 2024 Feb 10;14:3437. doi: 10.1038/s41598-024-53930-3 (PMC10858895; doi:10.1038/s41598-024-53930-3)
Supplement: Supplementary file 1 — Supplementary Information. [file 41598_2024_53930_MOESM1_ESM.pdf]

Manuscript title: The Influence of the COMT Val158Met Polymorphism on Prefrontal TDCS Effects on Aggression

Authors: Carmen Weidler, Lena Hofhansel, Christina Regenbogen, Dario Müller, Benjamin Clemens, Christian Montag, Andreas Reif, Ute Habel

Supplementary material

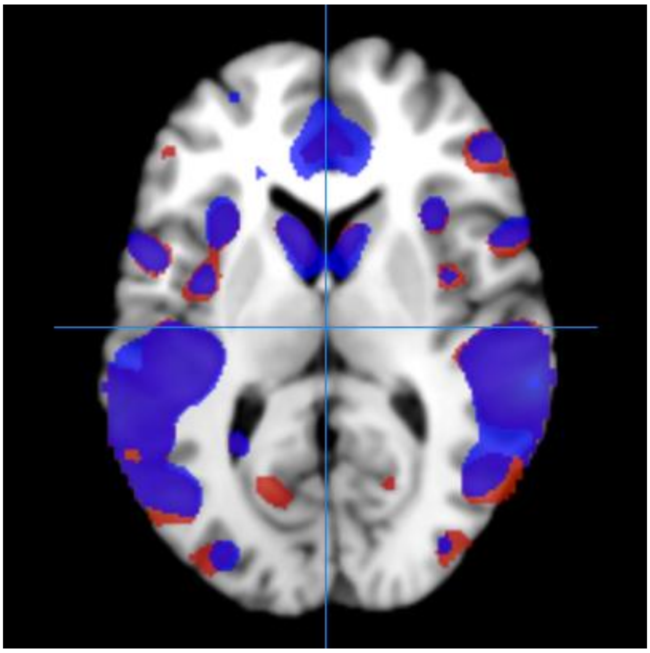

Supplementary figure S1: Average effect of the decision phase during the modified Taylor Aggression Paradigm. The image depicts the overlap of activation before (red) and after (blue) a single session of either sham or active transcranial direct current stimulation. Clusters extend over the right and left superior temporal gyrus, precuneus, insula, caudate nucleus, anterior cingulate cortex and mid orbital gyrus. *FWE* cluster-level correction(p<0.05).

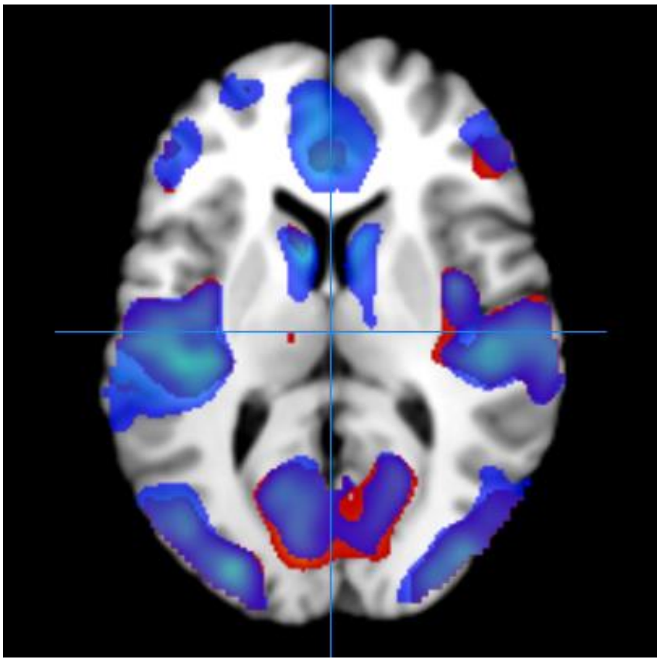

Supplementary figure S2: Average effect of the provocation phase during the modified Taylor Aggression Paradigm. The image depicts the overlap of activation before (red) and after (blue) a single session of either sham or active transcranial direct current stimulation. Clusters extend over the right and left inferior frontal gyrus, anterior cingulate cortex, lingual gyrus, fusiform gyrus, insula, right thalamus, left hippocampus, caudate and superior frontal gyrus. *FWE* cluster-level correction($p<0.05$).
